# Enterprise resilience to the COVID-19 pandemic: The role of business environment

**DOI:** 10.1371/journal.pone.0288722

**Published:** 2023-08-04

**Authors:** Lin Fu, Yupeng Shi, Xuze Yang, Wentao Zhang

**Affiliations:** 1 School of Economics, Central University of Finance and Economics, Beijing, China; 2 China Center for Internet Economy Research, Central University of Finance and Economics, Beijing, China; University of Agriculture Faisalabad, PAKISTAN

## Abstract

Enterprise resilience captures enterprises’ ability to survive and develop under uncertainties and shocks. Taking the shock of COVID-19 as an example, this paper employs a unique firm-level national survey dataset collected from a sample of nearly 40,000 Chinese private enterprises in 2020 to systematically explore the relation between local business environment and enterprise resilience. Our estimation results using ordered probit model indicate that favorable business environment can significantly enhance enterprise resilience and mitigate the shock of COVID-19 on firm performance. Mechanism analysis further shows that enterprises under better business environment generally have greater resilience as the result of timely and effective government support, reliable supply of production factors and logistics service, and lower levels of financial constraints. Our study deepens the understanding of the economic consequences of business environment and also sheds a new light on enterprise resilience enhancement.

## 1. Introduction

The sudden Covid-19 outbreak in 2020 brought not only panic to people but also unpredictable negative effects to enterprises. Although almost all enterprises have suffered severe losses during the pandemic, there are still distinct differences in the degree of loss across different enterprises, which are largely determined by their resilience [1]. The concept of "resilience" in economic research often associates with disaster management, which captures the ability of economies to make rapid adjustments and resume growth after suffering exogenous shocks [2]. As the micro-foundation of a country’s economic resilience, enterprise resilience reflects the enterprise’s ability to adjust to and recover from unexpected shocks and destructive events [3, 4]. Enterprises with stronger resilience can faster adjust and adapt to shocks and return to normal during crises, with smaller losses and greater chances of survival [5]. In the recent complex and changeable global environment, enhancing economic resilience has been the urgent desire of both economies and enterprises to deal with increasing uncertainty and instability, which highlights the significance of investigating the determinants of enterprise resilience in the context of large disruptive incidents.

According to existing literature, the determinants of enterprise resilience can be summarized as the following internal and external aspect. On one hand, some literature demonstrate that various internal characteristics of enterprises lead to the differences in their resilience. Those internal characteristics include the level of risk management and strategic decision-making [6], the ability of learning from past crises [7], firm size [8], innovation ability [9], social responsibility [10]. On the other hand, some literature argue that the external environment of enterprises plays a crucial role in the formation of enterprise resilience. Those external factors include the supply chain of production factors [11], labor supply and employment costs [12], transport and logistics service [13], financial support and other external assistance [14]. However, previous literature still have certain limitations. For example, although several external factors has been studied individually, few studies have taken the relation between enterprise resilience and overall local institutional environment into account.

The indicator “business environment”, usually applied to comprehensively measure the quality of local institutional environment for enterprise development, has been viewed as an essential manifestation of the soft power of a region for economic development. In recent China, optimizing business environment has become an essential part of deepening reform and one of the most critical works of local governments to attract external investments. In October 2019, the Chinese State Council officially issued the Regulation on Optimizing the Business Environment, which provided a strong guarantee and support for improving business environment at the legal level. Benefiting from the increasing government attention and various reform measures, China’s overall business environment has been significantly rising from 85th in 2017 to 31th in 2019 in the global ranking released by the World Bank. Research has shown that a good business environment significantly facilitates enterprise performance regarding saving operation costs [15], promoting innovation [16] and export [17], etc. According to economic theory, business environment has the potential to strengthen enterprise resilience. In general, enterprises under good business environment tend to be more likely to have good external conditions for growth, have more chances to obtain supporting policies and factor guarantees which are essential for survival and recovery under negative shocks. However, the assessment of the effects of local business environment on enterprise resilience has been largely neglected in previous empirical studies.

This paper aims to investigate whether the local business environment can enhance enterprise resilience to the COVID-19 epidemic. Based on a sample of nearly 40,000 private enterprises in the survey of “Tens of thousands of private enterprises evaluating the business environment” conducted by the All-China National Federation of Industry and Commerce (ACFIC) in 2020, our empirical results show that favorable business environment can significantly enhance enterprise resilience and mitigate the shock of COVID-19 on firm performance. Further mechanism analysis shows that enterprises under better business environment generally have greater resilience as the result of timely and effective government support, reliable supply of production factors and logistics service, and lower levels of financial constraints. Nowadays as most economies are stepping into the post-pandemic era, strengthening the construction of the business environment is conductive to the bounce back and sustainable development of enterprises.

Our paper contributes to the literature in several ways. First, this paper firstly employs large-scale enterprise survey data to empirically analyze the impact of local business environment on enterprise resilience. The existing literature suggests that external environment relates to enterprise performance, but it is not clear whether business environment affects enterprise resilience. Our study presents fresh evidence that good business environment can benefit enterprises from bailout policy, factor supply and financial support against the epidemic shock, which in turn enhances enterprise resilience. Second, this paper contributes to our knowledge of the economic consequences of business environment. Previous studies mainly focused on the impacts of business environment on firm performance under normal conditions (e.g., operating costs, innovation), while our study complements these by examining the effects of business environment on enterprise resilience during the unusual times. Third, our findings add to a growing body of evidence on the determinants of enterprises resilience to crises. By indicating that cross-city differences in business environment shape differences in enterprise resilience during the pandemic, we identify a new determinant of enterprises resilience that is particularly adapted to the COVID-19 shock.

## 2. Literature review

The literature closely related to the topic of this paper mainly includes the following two aspects:

### 2.1 The connotation of the business environment and its impact on enterprises

The business environment is the integration of all external environments for the operation and development of market entities, involving various dimensions such as public services, human resources, market environment, innovation environment, financial services, the rule of law environment and government affairs environment, which has a vast impact on market entities in the region [18]. In specific measurements, existing studies have quantified the regional business environment mainly based on the objective indicator and subjective evaluation method. The objective indicator method calculates the regional business environment score by selecting the local objective economic indicators for weighting. In contrast, the subjective evaluation method primarily evaluates the business environment according to the subjective perception of the respondents by conducting questionnaires on the market subjects in the region [19]. In recent years, empirical research on the impact of the business environment on enterprises has made many valuable research results. First, the existing literature shows that the business environment significantly impacts entrepreneurs’ confidence and behavior: optimizing the business environment can substantially improve entrepreneurs’ confidence, thereby promoting the high-quality development of enterprises [20]. Improving the business environment will help entrepreneurs reduce non-productive time, such as public relations and entertainment, allocate more time to enterprise production and operation, and reduce rent-seeking and apportionment expenses [21]. In addition, a good business environment can provide crucial external support for entrepreneurial behavior [22].

In addition, the business environment also impacts many aspects, such as enterprise exports, costs, and performance. In terms of exports, local governments can significantly improve the export performance of local enterprises by enhancing the local business environment (such as customs clearance efficiency, infrastructure, etc.) [17, 23]. In terms of cost, research shows that improving the business environment can reduce the enterprise’s institutional transaction cost [15] and financing costs by reducing the enterprise’s credit risk and improving its internal control quality [24]. In terms of business performance, the government’s policy of optimizing the business environment, represented by the reform of "decentralization, regulation, and service," has significantly promoted the business performance of enterprises [25]. In terms of innovation, a good business environment can promote enterprises to adopt more active innovation strategies and improve their innovation performance [16, 26]. It is optimizing the business environment by breaking the original rent-seeking mechanism, improving the flow and accuracy of public information, improving market efficiency, and enhancing the vitality and competitiveness of private enterprises [27]. In terms of investment, hosts with better market conditions are more likely to avoid investment risks, which is more conducive to attracting foreign investment [28]. Attracting foreign investment also helps to improve the host market environment and form a virtuous cycle [29].

### 2.2 The connotation of enterprise resilience and its influencing factors

Enterprise resilience is an important indicator reflecting the performance of enterprises during the crisis, reflecting the ability of enterprises to withstand the impact of the external environment and sustainable development. Enterprise resilience is an adaptive ability of an enterprise, which enables the enterprise to respond reasonably, adapt to the new environment and gradually recover after a business interruption [3, 4]. Enterprise resilience reflects the enterprise’s ability to reconfigure, recombine and reorganize its resources to cope with changes in the external environment, more stable stakeholder relationships, proactive risk management capabilities, and faster recovery to expected performance levels (such as inventory, capacity, service efficiency) [30]. As for the measurement of enterprise resilience, one approach is to summarize the main dimensions of enterprise resilience and synthesize the resilience index through research and interview on enterprises [31]. The other method is based on the changes in enterprise financial indicators (such as financing quota, employment, and investment efficiency) or market indicators (such as enterprise stock price and stock price volatility) before and after the crisis [32].

Regarding the determinants of enterprise resilience, the current literature mainly focuses on the following aspects of the impact on enterprise resilience. First, a stable supply of production factors. The production and operation of enterprises need a steady supply chain to ensure the supply of raw materials, intermediate products, water, electricity, heat, and other production factors. Especially in this epidemic, the enterprise’s factor supply has withstood a severe test [33]. Therefore, whether the production factors can be guaranteed stably under the impact of the epidemic determines the enterprise’s resilience to a large extent [11]. Second, the employment status of enterprises. Labor is an essential factor in determining resilience [12]. If the employability and willingness of labor are disturbed, it will harm enterprises’ employment and thus affect enterprises’ resilience [34, 35]. Third, the logistics are smooth. Whether raw materials and intermediate products from the upstream of the industrial chain or products sent downstream, they must be transported through logistics. Therefore, logistics has an important impact on enterprise resilience [13]. Fourth, the cash flow status of the enterprise. The enterprise cash flow management runs through production and operation, and the enterprise’s financing ability and financing cost will significantly affect the enterprise’s resilience [14]. Finally, external assistance will also have an impact on enterprise resilience. There is some evidence that external aid harms the resilience of enterprises. For example, federal disaster relief assistance funds will significantly damage the resilience of urban and rural enterprises [36]; However, some studies believe that external assistance can effectively improve enterprise resilience, such as insurance and mitigation measures can significantly reduce disaster losses and accelerate recovery [37]. In addition, some factors will affect the resilience of enterprises, such as enterprise size [8], innovation ability [9], social capital [38], digital ability [39], etc.

Based on the above literature, although the impact of the business environment on enterprise behavior and performance has been comprehensively studied, there are few studies focusing on the effects of the business environment on enterprise resilience. Moreover, previous literature on the determinants of enterprise resilience also neglected the role of the business environment in enterprises’ adapt to disruptions and recover from shocks. Thus it is worthwhile to use large-scale firm-level data to investigate the relation between business environment and enterprise resilience.

## 3. Data, variables and models

### 3.1 Data source

The primary data source of this paper is the survey data of "Tens of Thousands of Private Enterprises Evaluating Business Environment" conducted by the All-China National Federation of Industry and Commerce (ACFIC) in 2020. The survey collected the scores of the enterprises on the local factor environment, the rule of law environment, the government environment, the market environment, and the innovation environment, the understanding of the existing shortcomings, and the suggestions for future improvement. It made statistics on the operating conditions of the enterprises in the current year. In 2020, the survey covered 355 prefecture-level cities, county-level cities under provincial jurisdiction, and autonomous prefectures nationwide, and a total of 40,216 valid questionnaires were collected from enterprises. Excluding the samples which lack information about the enterprise registration place and the main business, we obtain 39,389 valid samples. We also collect urban-level macro data as control variables from China Urban Statistical Yearbook.

### 3.2 Variable description

#### 3.2.1 Explanatory variable

The explanatory variable of this paper is enterprise resilience. According to the research theme and data availability, this paper is engaged in the middle and post-epidemic dimensions. It measures enterprise resilience by two objective quantitative indicators, namely, the business status of the enterprise during the impact of the epidemic and the recovery speed after the first round of the epidemic outbreak. This paper uses the business status of enterprises in the first half of 2020 to measure the impact on enterprises. The answers to this question include "substantial loss," "slight loss," " break-even," "slight profit," and "substantial profit." This paper sets the dummy variable *Status* and assigns the above answers—2, - 1, 0, 1, and 2 points, respectively. The higher the score, the better the business performance and the stronger the enterprise’s resilience under the epidemic’s impact. The answer to the profit margin in this questionnaire is based on the subjective judgment of entrepreneurs. To avoid the subjective bias, we will simplify the explanatory variable as whether the enterprise has lost money in the first half of 2020, set the dummy variable *Status2*, assign 0 to the enterprises that answer "substantial loss" and "slight loss," and assign 1 to the enterprises that answer " break-even," "slight profit" and "substantial profit."

For the recovery speed, this paper uses two methods to measure it: one is the recovery speed of enterprise capacity or business, and the other is the speed of employee return to work. This paper adopts the enterprise’s answers to the "capacity utilization rate (resumption rate)" and "employee return rate" in the questionnaire and sets the virtual variable capacity utilization rate (*Utilization*) and employee return rate (*Resumption*) for five answers: "0–10%", "10% - 20%", "20% - 50%", "50% - 80%", and "more than 80%". The corresponding assigned values are 1–5 points. The higher the score, the faster the enterprise recovers.

#### 3.2.2 Explained variables

This paper focuses on whether the business environment can enhance enterprise resilience to the COVID-19 pandemic. Therefore, we take the natural logarithm of the cumulative number of confirmed COVID-19 cases at the municipal level by the end of June 2020 plus one to measure the severity of the local epidemic, recorded as *Lncase*. We focus on the cumulative confirmed cases until June 30 because the survey collected the business status of enterprises in the first half of 2020. The measurement of business environment is based on the enterprise’s answer to "Please evaluate the overall environment of the city’s business environment”, with a score varying from 1 to 5 points. Then we use the average of the scores from all enterprises at the municipal level as the measure of the city’s local business environment and assigned to all enterprises in the city, recorded as the *Environment*. We use the average score of firms located in the same city rather than the individual firm’s score to measure business environment because the city-level local business environment should be more objective and reliable. Averaging can reduce the individual bias caused by the subjective perception of each enterprise, which is also in line with previous studies [20, 40]. To capture the role of the business environment in mitigating the epidemic shock, we multiply the overall average score of the local business environment and the cumulative number of confirmed local COVID-19 cases as *BusXlncase*, which is the core variable of this paper.

#### 3.2.3 Control variables

First, we control the enterprise-level characteristics, including ① *Revenue*. Large-scale enterprises are less impacted and more resilient in this epidemic [41]. In this paper, we use two methods to measure the size of an enterprise: business income and the number of employees. According to the answers of the enterprises to the total revenue in 2019 in the questionnaire, and in combination with the Regulations on the Classification Standards of Small and Medium-sized Enterprises formulated by the Ministry of Finance, this paper divides the total annual revenue of enterprises into four levels: less than 5 million yuan, 5–30 million yuan, 30–100 million yuan and more than 100 million yuan, generating corresponding dummy variables. ② Number of employees of the enterprise (*Staff*). According to the answers of the enterprise to the number of employees in 2019 in the questionnaire, this paper divides the number of employees into three levels: less than 100, 100–300, and more than 300, and generates corresponding dummy variables. ③ Whether it is a foreign trade or going global enterprise (*Trade*). On the one hand, export diversification can smooth the demand fluctuation in the external market and help enterprises resist risks [42]. On the other hand, export-oriented enterprises face a more complex external economic and trade environment, which may be subject to a greater exogenous impact [43]. According to the answers to the questionnaire, if the enterprise is a foreign trade enterprise, a going global enterprise, or both, the value is assigned as 1; otherwise, the value is assigned as 0. ④ R&D investment proportion (*R&D*). Innovation helps to enhance enterprise resilience. Enterprises with greater investment in innovation have a stronger ability to cope with supply chain disruption crisis [44] and recover from the economic crisis faster [45]. This paper sets corresponding dummy variables according to the enterprise’s answer to "Please tell me the proportion of your R&D investment in total revenue in 2019", which are "below 1%", "1.1% - 5%", "5.1% - 7%", "7.1% - 10%" and "above 10.1%".

Second, we control the urban-level social and economic conditions. Since COVID-19 broke out nationwide in February 2020, which is relatively close to the end of 2019, the urban-level control variables in this paper are collected in 2019, including ① Population size (*Lnpop*). Cities with high population concentration have strong impact resistance, more robust economic recovery ability, and higher urban resilience [46]. Enterprises located in highly resilient cities may also have strong resilience, so this paper controls the average population of the enterprise location in 2019 and conducts logarithmic processing. ② Economic level (*Averagegdp*). Past studies have confirmed that there is a positive correlation between per capita GDP and urban resilience [47]. This paper measures the level of urban economic development by the per capita GDP of the city where the enterprise was located in 2019. ③ Fiscal expenditure level (*Fiscal*). Considering that the level of financial expenditure reflects the activity of local governments in economic activities, local governments with higher spending levels will also play a more critical role in the prevention and control of epidemic diseases, and previous research on resilience also controls the level of financial expenditure [48]. We measure the level of financial expenditure as the proportion of financial payments in local GDP in 2019. ④ Urban prevention and control capability (*Doctor*). The situation of medical resources will affect the local ability to prevent and control the epidemic, proxied as the number of doctors per 10,000 people in 2019.

In addition, we also control the fixed effect of the province where the city is located and the fixed effect of the industry to which the enterprise’s primary business belongs, which are recorded as (*Province*) and *(Industry*), respectively.

#### 3.2.4 Descriptive analysis

**Fig 1:**
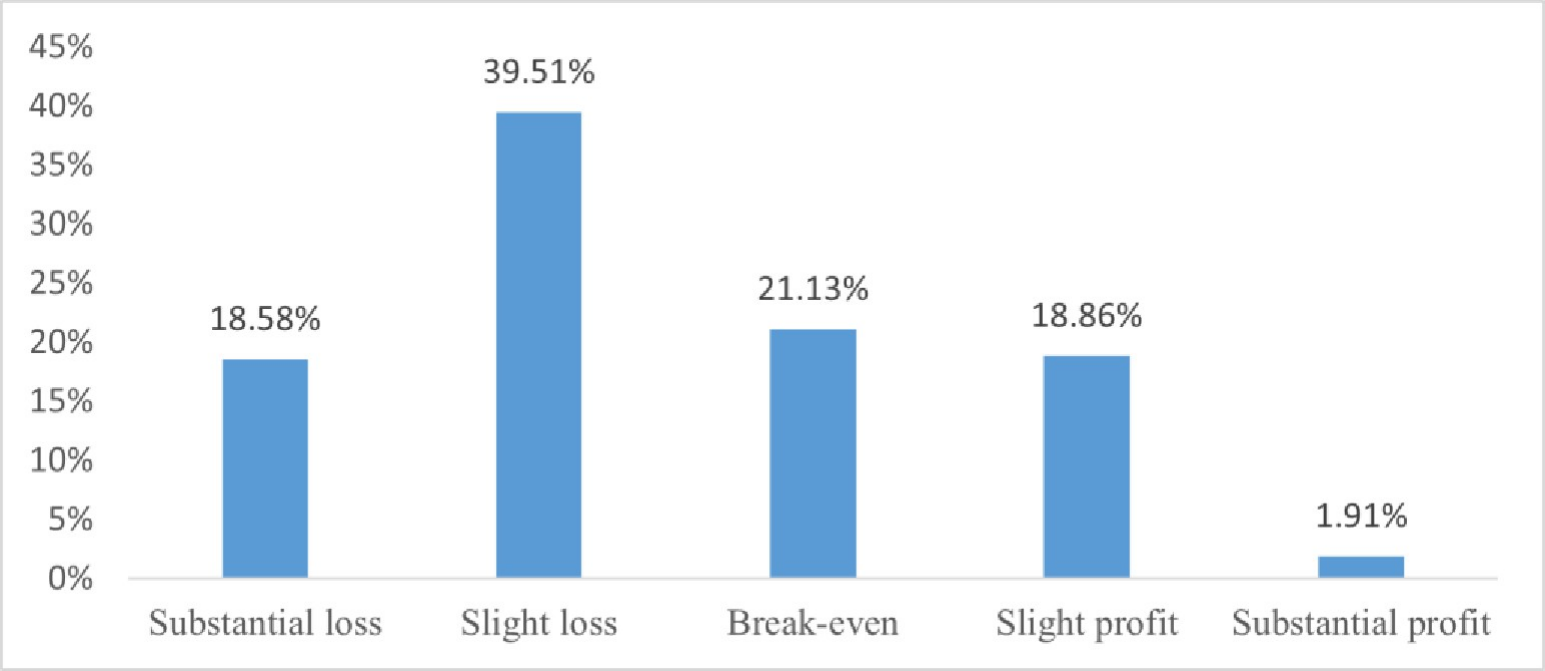
Overview of enterprises’ business status in the first half of 2020.

Fig 1 shows the statistics of the operating status of the sample enterprises in the first half of 2020. Most enterprises were in poor operating conditions, with nearly 60% losing money and only 20% making profits. The negative impact of COVID-19 to economy is pretty obvious.

Table 1 reports the descriptive statistics. It shows that the average score of the business environment faced by the sample enterprises is 4.03, which reflects the efforts of the local governments in China made to improve the business environment in recent years. The sample covers enterprises of all sizes, which can precisely reflect the overall situation of enterprises in China.

**Table 1:** Descriptive statistics.

*Variables*	*Obs*.	*Mean*	*SD*	Min	Max
*Environment*	39,389	4.03	0.27	1	5
*Lncase*	39,389	1.67	0.80	0	4.70
*BusXlncase*	39,389	6.76	3.25	0	17.23
*Status*	39,389	-0.54	1.05	-2	2
*Status2*	39,389	0.42	0.49	0	1
*Utilization*	39,389	3.66	1.36	1	5
*Resumption*	39,389	4.35	1.10	1	5
*Staff1*	39,389	0.62	0.49	0	1
*Staff2*	39,389	0.21	0.41	0	1
*Staff3*	39,389	0.17	0.38	0	1
*Revenue1*	39,389	0.39	0.49	0	1
*Revenue2*	39,389	0.31	0.46	0	1
*Revenue3*	39,389	0.17	0.37	0	1
*Revenue4*	39,389	0.14	0.35	0	1
*Rd1*	39,389	0.36	0.48	0	1
*Rd2*	39,389	0.32	0.47	0	1
*Rd3*	39,389	0.18	0.38	0	1
*Rd4*	39,389	0.08	0.27	0	1
*Rd5*	39,389	0.06	0.24	0	1
*Lnpop*	37,018	6.29	0.77	3.16	8.04
*Averagegdp*	37,018	78359.19	42539.55	14746.00	203489.00
*Fiscal*	37,018	20.84	11.63	6.62	148.75
*Doctor*	37,018	30.22	8.74	13.24	64.24

### 3.3 Empirical model

According to the discrete and ordered value type of our explained variable, this paper primarily uses the ordered probit model for empirical analysis. This discrete model has been widely applied in the analysis of scoring questionnaires [49]. The model specifications are as follows:

$$y_{ij}^{*}=\beta_{0}+\beta_{1}Lncase_{j}+\beta_{1}BusXlncase_{j}+\varphi \prime X_{ij}+Industry_{i}+Province_{j}+\varepsilon_{ij}$$ (1)

$$y_{ij}=\left\{ \begin{array}{l} -2\;y_{ij}^{*}\leq a_{1}\\ -1\;a_{1}<y_{ij}^{*}\leq a_{2}\\ \;0\;a_{2}<y_{ij}^{*}\leq a_{3}\\ \;1\;a_{3}<y_{ij}^{*}\leq a_{4}\\ \;2\;a_{4}<y_{ij}^{*} \end{array}\right.$$

where $y_{ij}^{*}$ is a potential variable that cannot be directly observed. *y_ij_* represents our explained variables of enterprise resilience, namely *Status*_*ij*_, *Utilization*_*ij*_, and *Resumption*_*ij*_, which capture the business status, capacity utilization, and employee return rate of enterprise *i* located in city *j* in the first half of 2020 respectively. In terms of the binary variable *Status2*_*ij*_, we switch to Probit model for estimation. *Lncase_j_* refers to the cumulative number of confirmed Covid-19 cases in the city where the enterprise is located by June 30, 2020. *BusXlncase_j_* represents the cross-multiplying term of the city-level average business environment score and *Lncase_j_. X_ij_* represents a series of control variables. *Industry_i_* represents the fixed effect of the industry of the enterprise i. *Province_j_* represents the fixed effect of the province where city *j* is located. *ε_ij_* is the residual item.

## 4. Results and discussion

### 4.1 Baseline regression

We report the baseline regression results in Table 2. In columns (1) and (2), the negative coefficients of *Lncase* reflect that the business status of the enterprise become worse under more severe epidemic shock, and in columns (3) and (4) the negative coefficients of *Lncase* imply that the recovery for enterprises become slower and harder under more severe epidemic shock. The coefficients of the cross-multiplying term *BusXlncase* are significantly and robustly positive, indicating that the business environment can indeed mitigate the epidemic shock on the operation of enterprises and thus enhance enterprise resilience. Regarding the scale of those coefficients, compared with business status and capacity utilization, employees’ return to work seems more sensitive to the epidemic shock and business environment. Among the control variables, foreign-trade enterprises perform better during the epidemic while their recovery speed is slower. Enterprises with higher business revenue tend to own better operating condition and faster recovery speed, consistent with previous findings that firm size and resilience are positively correlated. Enterprises with more employees can achieve better business status and capacity utilization during the epidemic, but also have more difficulties to make employees return to work.

**Table 2:** Baseline regression.

*Variables*	(1)	(2)	(3)	(4)
	*Status*	*Status2*	*Utilization*	*Resumption*
*Lncase*	-0.387***	-0.408***	-0.477***	-1.222***
	(0.068)	(0.080)	(0.067)	(0.078)
*BusXlncase*	0.010***	0.098***	0.134***	0.316***
	(0.017)	(0.020)	(0.017)	(0.020)
*Trade*	0.028*	0.055***	-0.272***	-0.540***
	(0.015)	(0.017)	(0.014)	(0.016)
*Staff2*	0.022	0.045**	0.135***	-0.033*
	(0.016)	(0.019)	(0.015)	(0.018)
*Staff3*	0.103***	0.159***	0.125***	-0.191***
	(0.019)	(0.022)	(0.018)	(0.020)
*Revenue2*	0.166***	0.162***	0.383***	0.356***
	(0.014)	(0.017)	(0.014)	(0.016)
*Revenue3*	0.314***	0.299***	0.585***	0.599***
	(0.018)	(0.021)	(0.018)	(0.020)
*Revenue4*	0.594***	0.552***	0.913***	1.087***
	(0.022)	(0.025)	(0.022)	(0.028)
*RD2*	0.036***	-0.018	0.046***	0.028*
	(0.014)	(0.017)	(0.015)	(0.017)
*RD3*	0.074**	0.075***	0.041*	-0.057***
	(0.017)	(0.020)	(0.017)	(0.019)
*RD4*	0.051*	0.032	0.048*	-0.024
	(0.023)	(0.027)	(0.021)	(0.025)
*RD5*	-0.003	-0.015	0.125***	0.154***
	(0.026)	(0.029)	(0.025)	(0.030)
*Urban control variables*	yes	Yes	yes	yes
*Provincial fixed effect*	yes	Yes	yes	yes
*Industry fixed effect*	yes	Yes	yes	yes
*Observations*	37,018	37,018	37,018	37,018

Note: Figures in brackets are robust standard errors; *, **, *** are significant at 10%, 5%, and 1%, respectively, and Tables 3 – 11 are the same.

### 4.2 Marginal effect analysis

The coefficient obtained through the ordered Probit model estimation cannot be directly used to measure the magnitude of the marginal effect. Thus we conduct further analysis to calculate the marginal effect of the business environment on enterprise resilience to the pandemic.

Table 3 reports the marginal effects of *Lncase* and *BusXlncase* on enterprises’ business status. The coefficients of *Lncase* in columns (1) and (2) are significantly positive, and the coefficients in columns (3)—(5) is significantly negative, indicating that the epidemic shock increases the probability of enterprises suffering losses, and reduces the probability achieving break-even or profits. Specifically, for each 1% increase in urban confirmed cases with other variables in the mean value, the probability of suffering substantial and slight losses increases by 9.8 and 4.6 percentage points respectively, and the probability of achieving break-even, slight profits, and substantial profits decreases by 3.9, 8.8 and 1.8 percentage points respectively. In general, the more serious the epidemic is, the more stringent the prevention measures taken by the government, and thus the more difficulties for enterprises lie ahead.

**Table 3:** Marginal effect analysis: Business status.

*Business status*	(1)	(2)	(3)	(4)	(5)
	*Substantial loss*	*Slight loss*	*Break-even*	*Slight profit*	*Substantial profit*
*Lncase*	0.098***	0.046***	-0.039**	-0.088***	-0.018**
	(0.017)	(0.008)	(0.007)	(0.016)	(0.003)
*BusXlncase*	-0.025***	-0.012**	0.010*	0.023**	0.005**
	(0.004)	(0.002)	(0.002)	(0.004)	(0.001)
*Enterprise control variables*	yes	yes	yes	yes	yes
*Urban control variables*	yes	yes	yes	yes	yes
*Provincial fixed effect*	yes	yes	yes	yes	yes
*Industry fixed effect*	yes	yes	yes	yes	yes
*Observations*	37,018	37,018	37,018	37,018	37,018

The coefficients of the intersection terms *BusXlncase* in columns (1) and (2) are significantly negative, and the coefficients of the intersection terms in columns (3)—(5) are significantly positive. Specifically, for the interaction of local business environment and the logarithm of confirmed cases increased by 1 with other variables given the mean value, the probability of enterprise suffering substantial losses and slight losses decreases by 2.5 and 1.2 percentage points, respectively, and the probability of enterprise achieving break-even, slight profits, and substantial profits increases by 1.0, 2.3 and 0.5 percentage points, respectively. The results imply that the business environment can significantly alleviate the epidemic’s negative impact and thus improve enterprises’ business status.

Tables 4 and 5 reports the marginal effects of *Lncase* and *BusXlncase* on enterprises’ capacity utilization and resumption of work, respectively. In Table 4, the coefficients of the logarithm of confirmed cases *Lncase* are significantly positive in Column (1)–(3) and negative in Column (5). Similarly in Table 5, the coefficients of the logarithm of confirmed cases *Lncase* are significantly positive in Column (1)–(4) and negative in Column (5). Results show that the shock of COVID-19 epidemic increases the probability of slow resumption of production and reduces the probability of rapid recovery for enterprises. Specifically, when other variables are taken the mean value, for every 1% increase in the number of confirmed cases, the probability that the enterprise’s capacity utilization rate (business resumption rate) is at “0–10%”, “10–20%”, and “20–50%” increases by 8.2, 4.7, and 3.5 percentage points respectively, and the probability that the utilization rate is at “above 80%” decreases by 16.4 percentage points; the probability of employees’ return-to-work rate at “0–10%”, “10–20%”, “20–50%” and “50% - 80%” increases by 10.1, 6.9, 8.9 and 12.4 percentage points respectively, and the probabilities of employees’ return-to-work rate at “above 80%” decreases by 38.3 percentage points. The results indicate that the epidemic shock has significantly reduced enterprise production capacity’s utilization rate and prevented employees from returning to work.

**Table 4:** Marginal effect analysis: Capacity utilization.

*Utilization*	(1)	(2)	(3)	(4)	(5)
	0–10%	10–20%	20–50%	50–80%	80%-100%
*Lncase*	0.082***	0.047***	0.035***	-0.001	-0.164***
	(0.012)	(0.007)	(0.005)	(0.001)	(0.023)
*BusXlncase*	-0.023***	-0.013***	-0.010***	0.0003	0.046***
	(0.003)	(0.002)	(0.001)	(0.0001)	(0.006)
*Enterprise control variables*	yes	yes	yes	yes	yes
*Urban control variables*	yes	yes	yes	yes	yes
*Provincial fixed effect*	yes	yes	yes	yes	yes
*Industry fixed effect*	yes	yes	yes	yes	yes
*Observations*	37,018	37,018	37,018	37,018	37,018

**Table 5:** Marginal effect analysis: Resumption of work.

*Resumption*	(1)	(2)	(3)	(4)	(5)
	0–10%	10–20%	20–50%	50–80%	80%-100%
*Lncase*	0.101***	0.069***	0.089***	0.124***	-0.383***
	(0.007)	(0.005)	(0.006)	(0.008)	(0.024)
*BusXlncase*	-0.026***	-0.018***	-0.023***	-0.032***	0.099***
	(0.002)	(0.001)	(0.001)	(0.002)	(0.006)
*Enterprise control variables*	yes	yes	yes	yes	yes
*Urban control variables*	yes	yes	yes	yes	yes
*Provincial fixed effect*	yes	yes	yes	yes	yes
*Industry fixed effect*	yes	yes	yes	yes	yes
*Observations*	37,018	37,018	37,018	37,018	37,018

In Table 4, the coefficients of the interaction term *BusXlncase* are significantly negative in columns (1)–(3) and is positive in columns (5). Similarly in Table 5, the coefficient of the interaction term *BusXlncase* are significantly negative in columns (1)–(4) and positive in columns (5). Specifically, when other variables are taken the mean value, for the interaction of local business environment and the logarithm of confirmed cases increased by 1, the probability of the enterprise’s capacity utilization rate (business resumption rate) at “0–10%”, “10–20%”, and “20–50%” decreases by 2.3, 1.3, and 1 percentage points, respectively, and the probability of being “above 80%” increases by 4.6 percentage points; the probability of the return-to-work rate of employees at “0–10%”, “10–20%”, “20–50%”, and “50% - 80%” decreases by 2.6, 1.8, 2.3, and 3.2 percentage points respectively, and the probability of being “above 80%” increases by 9.9 percentage points. Those results demonstrate that local business environment exerts mitigating effects on the negative shocks of epidemic, promoting enterprise capacity utilization and facilitating the employees return to work.

### 4.3 Robustness checks

#### 4.3.1 Replacing the measure of business environment

In the baseline regression, this paper measures the local business environment as the average value of the business environment scores of all enterprises in the same city. In the robustness test, we multiplies each enterprise’s score of the business environment evaluation and the number of confirmed cases in the city as an alternative measure of business environment, recorded as *BusXlncase2*. The regression results shown in Table 6 are robust and consistent with the baseline results.

**Table 6:** Robustness test: Alternative measure of business environment.

*Variables*	(1)	(2)	(3)	(4)
	*Status*	*Status2*	*Utilization*	*Resumption*
*Lncase*	-0.150***	-0.184***	-0.252***	-0.343***
	(0.025)	(0.029)	(0.024)	(0.027)
*BusXlncase2*	0.038***	0.040***	0.076***	0.087***
	(0.004)	(0.004)	(0.003)	(0.004)
*Enterprise control variables*	yes	Yes	yes	yes
*Urban control variables*	yes	Yes	yes	yes
*Provincial fixed effect*	yes	Yes	yes	yes
*Industry fixed effect*	yes	Yes	yes	yes
*Observations*	37,018	37,018	37,018	37,018

#### 4.3.2 Replacing the measure of epidemic shock

The epidemic’s shock on the economy includes two aspects. On the one hand, the epidemic affects the health of residents and the labor supply of economic activities. On the other hand, the government will take countermeasures such as lockdown to control the spread of the epidemic, which also harms the economy. Therefore, in the robustness test, this paper alternatively measures the shock of the epidemic by the number of emergency response days for major public health emergencies [50].

According to the National Overall Emergency Plan for Public Emergencies, there are four levels of emergency response for major public health emergencies in China, and the response intensity from Level I (especially significant) to Level IV (general) gradually decreases with the State Council, provincial government, municipal government, and county government as the primary initiators. On January 23, 2020, 31 provinces in China launched Level I response to major public health emergencies and gradually lowered it to Level II and Level III emergency response after a while. Considering that under the level III emergency response, the epidemic prevention and control measures have limited impact on economic activities, this paper mainly considers level I and level II responses. We alternatively summarize the number of Level I response days experienced by the city multiplied by two and the Level II response days and then divide it by 159 (159 days from January 23 when the emergency response started to June 30) to measure the impact of epidemic. We define the alternative measure of epidemic shock as *Emergency* and the cross-multiplying term between the business environment and *Emergency* as *BusXemergency*. The regression results shown in Table 7 are consistent with the baseline results.

**Table 7:** Robustness test: Alternative measure of epidemic shock.

*Variables*	(1)	(2)	(3)	(4)
	*Status*	*Status2*	*Utilization*	*Resumption*
*Emergency*	-1.537***	-1.450***	-1.122***	-2.213***
	(0.296)	(0.357)	(0.242)	(0.286)
*BusXemergency*	0.317***	0.341***	0.368***	0.671***
	(0.038)	(0.046)	(0.038)	(0.044)
*Enterprise control variables*	yes	Yes	yes	yes
*Urban control variables*	yes	Yes	yes	yes
*Provincial fixed effect*	yes	Yes	yes	yes
*Industry fixed effect*	yes	Yes	yes	yes
*Observations*	37,018	37,018	37,018	37,018

#### 4.3.3 Removing the sample of Hubei Province

As the province where the Covid-19 first outbroke in China, Hubei was affected severely by the epidemic at the beginning of 2020. The number of confirmed cases and the time of lockdown in Hubei were far more than those in other provinces at that time. To exclude the particular impact of Hubei Province, we remove the sample of Hubei Province and conduct regression again. The results shown in Table 8 remain robust and stable.

**Table 8:** Robustness test: Removing the sample of Hubei.

*Variables*	(1)	(2)	(3)	(4)
	*Status*	*Status2*	*Utilization*	*Resumption*
*Lncase*	-0.490***	-0.580***	-0.561***	-1.311***
	(0.081)	(0.096)	(0.083)	(0.094)
*BusXlncase*	0.122***	0.136***	0.153***	0.336***
	(0.019)	(0.023)	(0.020)	(0.023)
*Enterprise control variables*	yes	Yes	yes	yes
*Urban control variables*	yes	Yes	yes	yes
*Provincial fixed effect*	yes	Yes	yes	yes
*Industry fixed effect*	yes	Yes	yes	yes
*Observations*	35,346	35,346	35,346	35,346

#### 4.3.4 Removing cities with less than 50 enterprises

To avoid the disturbance of extreme values of individual enterprises on our results, we remove cities with a sample of less than 50 enterprises and conduct regression again. The results shown in Table 9 are still robust.

**Table 9:** Robustness test: Removing cities with less than 50 enterprises.

*Variables*	(1)	(2)	(3)	(4)
	*Status*	*Status2*	*Utilization*	*Resumption*
*Lncase*	-0.387***	-0.422***	-0.447***	-1.206***
	(0.070)	(0.083)	(0.069)	(0.080)
*BusXlncase*	0.099***	0.010***	0.126***	0.311***
	(0.017)	(0.020)	(0.017)	(0.020)
*Enterprise control variables*	yes	Yes	yes	yes
*Urban control variables*	yes	Yes	yes	yes
*Provincial fixed effect*	yes	Yes	yes	yes
*Industry fixed effect*	yes	Yes	yes	yes
*Observations*	35,379	35,379	35,379	35,379

### 4.4 Mechanism analysis

#### 4.4.1 Introducing efficient and timely policies

At the beginning of the outbreak of the COVID-19 epidemic, enterprise production was greatly impacted. Governments at all levels have issued a series of rescue and support policies, which have played a significant role in helping enterprises cope with the epidemic. In areas with a good business environment, the government has a good sense of service, and administrative efficiency is often higher. Therefore, it is possible to introduce and implement effective rescue and support measures more quickly, thereby improving the resilience of enterprises. This paper constructs the efficiency and timeliness of rescue and support policies with dummy variables based on the answers of enterprises to "the content of all kinds of rescue and support policies" and "the timeliness of all kinds of rescue and support policies" in the questionnaire. The value range is 1–5 points. The higher the score, the more influential the rescue and support measures will be. The regression results in Column (1)-(2) in Table 10 show that the business environment can speed up the government’s response to introduce effective relief support measures significantly and therefore enhance enterprise resilience. Our results are also in line with previous findings that government support is significant for enterprises to cope with risks such as natural disaster [51]. Local government policies can reduce the losses suffered by small and medium-sized enterprises by improving disaster prevention measures and thereby enhancing their resilience [52].

**Table 10:** Mechanism analysis (1).

*Variables*	(1)	(2)	(3)	(4)	(5)
	*Efficiency*	*Timeliness*	*Factorsupply*	*Laborsupport*	*Logistics*
*Lncase*	-1.428***	-1.620***	-1.760***	-1.473***	-1.520***
	(0.068)	(0.069)	(0.070)	(0.069)	(0.070)
*BusXlncase*	0.357***	0.409***	0.444***	0.374***	0.390***
	(0.017)	(0.017)	(0.017)	(0.017)	(0.017)
*Enterprise control variables*	yes	yes	Yes	yes	yes
*Urban control variables*	yes	yes	Yes	yes	yes
*Provincial fixed effect*	yes	yes	Yes	yes	yes
*Industry fixed effect*	yes	yes	Yes	yes	yes
*Observations*	37,018	37,018	37,018	37,018	37,018

#### 4.4.2 Providing reliable production guarantee

In the first half of 2020, the sudden epidemic significantly affected Chinese enterprises’ production. First, the epidemic caused a shortage of production factors and affected the supply chain [53]. Second, the epidemic restricted labor mobility and reduced employment willingness [54]. Finally, the epidemic weakened the transportation capacity of the logistics industry and hindered the sales of enterprises’ products. All the above factors have brought challenges to the sustainable operation ability of enterprises, thus weakening enterprise resilience. In terms of a favorable business environment, the government has a stronger sense of service, which can better ensure the smooth flow of production factors, labor supply, and logistics, thus enhancing enterprise resilience. This paper measures the ability to guarantee production factor supply with the enterprise’s "overall evaluation of factor resource guarantee", measures the ability to guarantee labor supply with the enterprise’s "evaluation of labor acquisition and guarantee" and measures the ability to ensure smooth logistics with the enterprise’s "evaluation of logistics facilities and guarantee". The dummy variables *Factorsupply*, *Laborsupport*, and *Logistics* are set respectively, with a value range of 1–5 points. The higher the score is, the stronger the guaranteed ability is.

The regression results in Column (3)-(5) in Table 10 show that the epidemic’s impact has significantly damaged the supply chain, employment and logistics support capabilities of enterprises, and the business environment can significantly relieve this negative impact, thereby guaranteeing the sustainable operation of enterprises and enhancing their resilience.

#### 4.4.3 Relieving financing constraints of enterprises

The epidemic’s effect has caused the production and operation of many enterprises in China to stagnate, and there is tremendous pressure on the cash flow of enterprises. According to a sample survey, 70% of SMEs can only maintain their cash flow for three months. Given a good business environment, the rule of law construction is also more perfect, the availability and quality of credit information are better, and enterprises are more likely to obtain financing from formal channels and face more relaxed financing constraints, thus enhancing enterprise resilience. Based on the answers of the enterprise to "What is the loan limit your company has obtained from the bank since January 2020?", "According to your understanding, what is the comprehensive annual interest rate of your company’s latest bank loan?" and "The difficulty of obtaining bank loans," the dummy variables *Amount*, *Rate*, and *Difficulty* are set as the explained variables for regression.

According to the regression results in Table 11, we find that the epidemic’s impact has significantly reduced the number of bank loans obtained by enterprises, increased the level of enterprise loan interest rates, and increased the difficulty for enterprises to get bank loans. The business environment helps mitigate the epidemic’s impact, relax the financing constraints enterprises face, and thus enhance the resilience of enterprises.

**Table 11:** Mechanism analysis (2).

*Variables*	(1)	(2)	(3)
	*Amount*	*Rate*	*Difficulty*
*Lncase*	-0.565***	0.218***	-1.196***
	(0.0822)	(0.0711)	(0.0667)
*BusXlncase*	0.149***	-0.0648***	0.304***
	(0.0204)	(0.0175)	(0.0166)
*Enterprise control variables*	yes	yes	yes
*Urban control variables*	yes	yes	yes
*Provincial fixed effect*	yes	yes	yes
*Industry fixed effect*	yes	yes	yes
*Observations*	20,911	31,181	37,018

## 5. Conclusion, policy implications and future research

The COVID-19 epidemic has brought many difficulties and challenges to the operation of enterprises, which largely determines the safety and prosperity of the economy. Using large-scale enterprise-level survey data in China to conduct systematic empirical research, this study indicates that a favorable local business environment can significantly enhance the resilience of enterprises and mitigate the shock of COVID-19 on firm performance, and this conclusion is consistent through a variety of robustness checks. Furthermore, this paper explores the mechanisms through which the business environment affects enterprise resilience in depth, and demonstrates that good business environment can enhance enterprise resilience to the COVID-19 pandemic through introducing efficient and timely policies, providing reliable production factor supply, and relieving financial constraints of enterprises.

Our paper represents a first step towards understanding the role of business environment in shaping enterprise resilience to the COVID-19 pandemic in a transition economy such as China, and our findings provide some policy implications on enterprise resilience enhancement during crises. Local governments should fully recognize the importance of business environment on enterprise resilience formation and regional economic stability. By creating an ideal business environment, the city can not only attract more enterprises and investments in normal times, but also obtain a stronger resilience to cope with economic uncertainties and maintain economic competitiveness under shocks or crises. Through improving business environment, enterprises especially small and medium-size firms can benefit from timely and effective bailout policies, reliable supply of production factors, and tangible financial support, which remarkably ease the pressure and difficulties of businesses and consolidate the foundation of economic recovery.

Despite our contributions to the literature, our study still contains limitations for future research. First, as running enterprises are complex and heterogenous, other factors related with resilience worth more attention, such as innovation capability and risk management. Second, the epidemic prevention and control period lasted for three years, but due to the availability of data, this paper only focuses on the first half of 2020 without cross-validation based on other periods. Finally, this paper mainly discusses resilience from a neutral and post-event perspective without discussing pre-event resilience.
